# A Proposal for a Composite with Temperature-Independent Thermophysical Properties: HfV_2_–HfV_2_O_7_

**DOI:** 10.3390/ma13215021

**Published:** 2020-11-07

**Authors:** Philipp Keuter, Anna L. Ravensburg, Marcus Hans, Soheil Karimi Aghda, Damian M. Holzapfel, Daniel Primetzhofer, Jochen M. Schneider

**Affiliations:** 1 Materials Chemistry, RWTH Aachen University, Kopernikusstr. 10, 52074 Aachen, Germany; anna.ravensburg@physics.uu.se (A.L.R.); hans@mch.rwth-aachen.de (M.H.); karimi@mch.rwth-aachen.de (S.K.A.); holzapfel@mch.rwth-aachen.de (D.M.H.); schneider@mch.rwth-aachen.de (J.M.S.); 2 Department of Physics and Astronomy, Uppsala University, Box 516, 75120 Uppsala, Sweden; daniel.primetzhofer@physics.uu.se

**Keywords:** thermoelasticity, negative thermal expansion, composites, magnetron sputtering

## Abstract

The HfV_2_–HfV_2_O_7_ composite is proposed as a material with potentially temperature-independent thermophysical properties due to the combination of anomalously increasing thermoelastic constants of HfV_2_ with the negative thermal expansion of HfV_2_O_7_. Based on literature data, the coexistence of both a near-zero temperature coefficient of elasticity and a coefficient of thermal expansion is suggested for a composite with a phase fraction of approximately 30 vol.% HfV_2_ and 70 vol.% HfV_2_O_7_. To produce HfV_2_–HfV_2_O_7_ composites, two synthesis pathways were investigated: (1) annealing of sputtered HfV_2_ films in air to form HfV_2_O_7_ oxide on the surface and (2) sputtering of HfV_2_O_7_/HfV_2_ bilayers. The high oxygen mobility in HfV_2_ is suggested to inhibit the formation of crystalline HfV_2_–HfV_2_O_7_ composites by annealing HfV_2_ in air due to oxygen-incorporation-induced amorphization of HfV_2_. Reducing the formation temperature of crystalline HfV_2_O_7_ from 550 °C, as obtained upon annealing, to 300 °C using reactive sputtering enables the synthesis of crystalline bilayered HfV_2_–HfV_2_O_7_.

## 1. Introduction

Volume expansion upon heating is probably the most prominent example of the influence of temperature on the physical properties of materials. However, for electronic, optical, and other high-precision devices, whose performances are critically affected by slight variations in volume, near-zero thermal expansion materials are desired [1,2,3,4,5]. Moreover, for mechanical components of precision instruments, temperature-independent volumes and elastic moduli are required [6,7,8]. A combination of both properties has only been obtained in gum metals [9,10] and Fe-Ni alloys [11] after intense deformation, thereby promoting the development of materials with intrinsic near-zero expansion and temperature-invariant elastic behavior irrespective of the plastic deformation route.

To compensate for thermally-induced volume changes, implementation of materials with negative thermal expansion (NTE) in a composite is a widely propagated approach [4,5,12]. Here, we propose to join a material with NTE properties with another exhibiting anomalously increasing thermoelastic constants in an attempt to obtain a combination of temperature-invariant elastic behavior and volume. This is a pioneering approach for the design of materials with temperature-independent thermophysical properties.

However, elastic constants commonly decrease monotonically with rising temperatures due to the anharmonicity of lattice vibrations [13,14]. Materials that deviate from this trend, thereby exhibiting anomalous thermoelastic behavior, constitute promising candidates for the design of composites with temperature-independent thermoelastic properties. While nowadays NTE has been observed in a wide variety of material families, e.g., zirconium tungstates and vanadates, zeolites, metal cyanides, metal-organic framework materials, perovskites, and anti-perovskites [12,15,16,17,18], reports of materials with anomalous thermoelastic behavior are scarce. The cubic transition metals V, Nb, Ta, Pd, and Pt each exhibit a thermoelastic anomaly in their shear elastic constants *c*_44_ [19,20,21,22,23]. Furthermore, binary Nb–Zr, Nb–Mo, Pd–Ag, and Pd–Rh solid solutions behave anomalously within well-defined concentration ranges [24,25,26]. Experimental and theoretical studies revealed the combination of a high density of states and electronic reallocation upon lattice distortion in the vicinity of the Fermi level to be the physical origin of the anomalous thermoelastic behavior [27,28,29,30,31]. However, the anomaly in these systems is mostly limited to the shear elastic constant *c*_44_, whereas the remaining elastic constants (there are three independent elastic constants for cubic symmetries, i.e., *c*_11_, *c*_12_, and *c*_44_) behave normally. Consequently, their thermoelastic anomaly is highly directionally dependent, but an isotropic temperature-independent behavior is desired for the proposed composite. Intermetallic cubic HfV_2_ (space group: Fm-3m), which is stable from around 112 K up to the melting point of 1820 K [32,33], exhibits increasing thermoelastic constants in all probed directions [32] so that an increase in the macroscopic elastic modulus (*E*) upon heating was measured in polycrystalline samples [34,35]. Thus, HfV_2_ is an ideal constituent to aim for a temperature-invariant elastic behavior in a composite. The second component, consequently, serves then for the compensation of the thermoelastic increase and for the positive thermal expansion (PTE) of HfV_2_. The linear coefficient of thermal expansion of HfV_2_ has been measured to be 9.9 × 10^−6^ K^−1^ around room temperature [36].

Multiple mechanisms may give rise to NTE [5,17], e.g., the magnetovolume effect, phase transitions, atomic radius contraction, and flexible network structures, whereas the latter is the prevalent physical origin in most NTE materials. In general, the expansion effect due to longitudinal vibrations in these flexible network structures is over-compensated by the contraction owing to transverse vibrations [5,37]. In regard to the search for suitable NTE materials, anisotropic contraction restricts their practical usability, so cubic phases, which exhibit inherently isotropic contraction, are particularly promising. In the group of flexible network materials, ZrW_2_O_8_ and isostructural HfW_2_O_8_ have an unprecedented isotropic NTE range of 0.3 to 1050 K [38,39]. Besides, the only ternary oxide within the Hf-V–O system, HfV_2_O_7_, exhibits isotropic NTE above approximately 370 K with a negative coefficient of thermal expansion of −7.2 × 10^−6^ K^−1^ [40]. With decreasing temperature, phase transformations first into an incommensurate structure, stable between around 369 and 340 K, and finally into a cubic 3 × 3 × 3 superstructure, are obtained [41]. Hence, forming a composite of HfV_2_ with the corresponding ternary oxide, HfV_2_O_7_, appears promising for tailoring the physical properties of the composite material towards thermal invariance.

Physical vapor deposition techniques have proven to be successful in synthesizing and refining materials with NTE properties [42,43,44,45]. Consequently, after assessing that a combination of both temperature-invariant elastic behavior and volume is achieved simultaneously for a certain phase fraction ratio, two synthesis pathways for HfV_2_–HfV_2_O_7_ composites were studied: (1) annealing of magnetron sputtered HfV_2_ thin films in air to form a HfV_2_O_7_ oxide scale on the thin film surface and (2) magnetron sputtering of HfV_2_O_7_ on HfV_2_. Furthermore, the NTE properties of HfV_2_O_7_ were verified for single-layered HfV_2_O_7_ thin films using temperature-dependent in situ X-ray diffraction.

## 2. Materials and Methods

### 2.1. Experimental Methods

Depositions were carried out by direct current magnetron sputtering at a target-to-substrate distance of 10 cm using elemental Hf and V (both 99.9% purity, 50 mm diameter) targets while the substrate remained at floating potential. For the synthesis of stoichiometric HfV_2_, the employed target power densities were 2.0 and 10.2 W cm^−2^ for Hf and V, respectively. Ar (99.9999%) was used as sputtering gas to achieve a working pressure of 0.4 Pa. The base pressure (at deposition temperature) was below 1 × 10^−6^ Pa. HfV_2_ films were deposited without intentional heating and at substrate temperatures of 500 and 700 °C. To ensure high purity of deposited HfV_2_, the following measures were taken: The backsides of the single-crystalline sapphire (0001) substrates were deposited beforehand with approximately 250 nm of Nb to overcome their partial transmissivity to radiation during heating. This consequently reduced the heat impact on the chamber walls, and thus, a considerable decrease in base pressure at elevated deposition temperatures was achieved. Second, prior to each deposition, all targets were sputtered for 5 min with closed shutters (positioned approximately 2 cm opposite of the target) to make the surfaces free from condensed impurities and to getter residual gases. Third, elemental Zr was additionally sputtered at 20 W against a shutter during all depositions to exploit its pronounced affinity for oxygen [46,47] as a getter pump to further reduce residual gas incorporation [48] into the growing HfV_2_ thin film. The Zr concentration in the as-deposited films was below 0.7 at.% based on energy-dispersive X-ray spectroscopy (EDX). Subsequently, selected films were capped after cooling to room temperature with an approximately 10 nm thick Nb layer to prevent impurity incorporation into the as-deposited thin film during air exposure.

HfV_2_O_7_ thin films were deposited at a substrate temperature of 450 °C in a reactive Ar/O_2_ (99.999%) atmosphere at a constant working pressure of 0.86 Pa while varying the O_2_ partial pressure between 0.05 and 0.13 Pa. Further information can be found elsewhere [49]. For the HfV_2_–HfV_2_O_7_ bilayer deposition, the synthesis procedure was the following: First, HfV_2_ was deposited at 700 °C for 75 min. Afterwards, the system was cooled down in vacuum to the synthesis substrate temperatures for HfV_2_O_7_, which were 250, 300, and 350 °C. Pure Ar was used for plasma ignition before O_2_ (p_O2_ = 0.09 Pa) was introduced to reactively sputter HfV_2_O_7_ for 100 min.

Annealing experiments were performed in a GERO F 40-200/13 air furnace (Carbolite Gero, Neuhausen, Germany). The Hf-V ratio in the synthesized films was measured by EDX carried out in a JEOL JFM-6480 SEM (JEOL Ltd., Tokyo, Japan) equipped with an EDAX Genesis 2000 device (EDAX Inc., Mahwah, NJ, USA) at an acceleration voltage of 20 kV. Chemical composition depth profiling of HfV_2_ was done by time-of-flight elastic recoil detection analysis (ToF-ERDA) at the Tandem Accelerator Laboratory of Uppsala University. 36 MeV ^127^|^8+^ primary ions and a time-of-flight telescope in combination with a Si solid-state detector for energy discrimination were used. Further details on the detector telescope can be found elsewhere [50]. The time and energy coincidence spectra were evaluated using the CONTES software package [51]. O and H depth profiles were characterized by ToF-ERDA, while the Hf-V ratio was obtained from EDX spectra. It should be noticed that Nb-capped und uncapped HfV_2_ were measured by ToF-ERDA under identical conditions, hence, systematic uncertainties do not affect this comparison. The structure of the synthesized films was studied using X-ray diffraction (XRD) carried out with a Bruker AXS D8 Discover General Area Detector Diffraction System (Bruker Corporation, Billerica, MA, USA). A Cu K_α_ source (current 40 mA, voltage of 40 kV) was used with a 0.5 mm pinhole collimator. Scans were performed at a fixed incidence angle of 15°. Selected samples were measured between room temperature and 475 °C using a DHS 1100 Hot Stage (Anton Paar, Ostfildern-Scharnhausen, Germany) equipped with a NI-NiCr thermocouple to measure the surface temperature. Peak fitting was conducted using TOPAS software (version 3) with a pseudo-Voigt II function. Lattice parameters of the cubic structures were consequently calculated employing Bragg’s law [52]. The lattice parameters of HfV_2_ were determined by averaging the data from (220), (311), (222), (331), (422), (333), and (044) reflections. Based on the obtained changes in lattice parameters with temperature, the linear coefficient of thermal expansion for cubic HfV_2_O_7_ was calculated by averaging the data from (200), (210), (211), (211), (220), (311), and (222) reflections. Standard deviations are added to evaluate the fitting quality. Morphology of bilayered HfV_2_–HfV_2_O_7_ was studied using scanning transmission electron microscopy (STEM) carried out in an FEI Helios Nanolab 660 dual-beam microscope (Thermo Fisher Scientific, Waltham, MA, USA). Cross-sectional sample preparation was conducted by focused ion beam techniques with a Ga^+^ source following a standard lift-out procedure [53].

### 2.2. Theoretical Methods

Ground state equilibrium lattice parameters for cubic HfO_2_ (Fm-3m, 12 atoms), VO_2_ (Fm-3m, 12 atoms), VO (Fm-3m, 8 atoms), and HfO (Fm-3m, 8 atoms) were calculated within the framework of density functional theory (DFT) [54] employing the Vienna ab initio simulation package (VASP) [55,56] with projector augmented wave potentials. The generalized gradient approximation, as introduced by Perdew, Burke, and Ernzerhof [57], was used for all calculations. Integration in the Brillouin zone was performed on a 20 × 20 × 20 *k*-point grid according to Monkhorst and Pack [58]. The total energy convergence criterion was 0.01 meV within a 500 eV cut-off. The equilibrium lattice parameters were determined by a Birch–Murnaghan equation of state [59,60] fit.

## 3. Results and Discussion

### 3.1. Composite Assessment

First, the suitability of HfV_2_–HfV_2_O_7_ as a composite with temperature-independent physical properties is evaluated, which relies on the coexistence of a near-zero coefficient of thermal expansion and a near-zero temperature coefficient of elasticity (*TCE*) for a certain phase fraction ratio. The *TCE* is defined by

(1) $$TCE\;=\;\frac{1}{E}\frac{dE}{dT}$$

and is estimated for HfV_2_–HfV_2_O_7_ composite using a rule-of-mixture approach (weighted average based on the volume fractions). While the temperature dependence of the elastic modulus of HfV_2_ is taken from the literature [35], no thermoelasticity data for HfV_2_O_7_ have been reported. The temperature-dependent elastic modulus has consequently been estimated from the experimentally obtained elastic modulus of HfV_2_O_7_ [49] assuming the same relative decline with temperature, as reported for the NTE material ZrW_2_O_8_ [61]. ZrW_2_O_8_ and HfV_2_O_7_ exhibit comparable negative coefficients of thermal expansion of −9.1 × 10^−6^ [62] and −7.2 × 10^−6^ K^−1^ [40], respectively, and share common structural features, both forming an openly-packed network structure of octahedral (Zr/Hf)O_6_ and polyhedral (W/V)O_4_ units connected by corner-sharing oxygen atoms [63]. The *TCE* of the composite was averaged over a temperature range from 120 (onset of NTE behavior in HfV_2_O_7_ [40,41,64]) to 300 °C.

**Figure 1:**
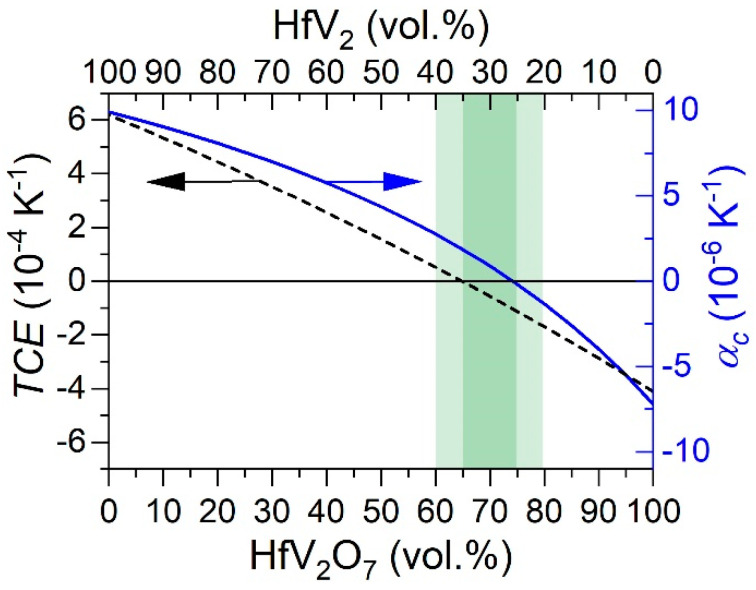
Variation of the temperature coefficient of elasticity *TCE* (black dashed line) and linear coefficient of thermal expansion $\alpha_{c}$ (blue solid line) of composite material HfV_2_–HfV_2_O_7_ depending on the volume fractions of the individual constituents. The region of interest is highlighted in green.

On the other hand, the coefficient of thermal expansion of a composite usually does not follow a simple rule-of-mixture behavior [65]. NTE materials are generally less stiff (lower *E*) than expected based on the bond strengths [66], and thus normally constitute the more compliant component in the composite. As a result, the elastic deformation during expansion and contraction is predominantly concentrated on the NTE component reducing its impact on the overall expansion coefficient. Consequently, theoretical models to describe the expansion coefficient of a composite typically also take the elastic properties of the individual components into account [66]. In the Turner model the overall coefficient of thermal expansion of the composite $\alpha_{c}$ is described by

(2) $$\alpha_{c}\;=\;\frac{\sum_{i}B_{i}\alpha_{i}\phi_{i}}{\sum_{i}B_{i}\phi_{i}},$$

where $B_{i}$, $\alpha_{i}$, and $\phi_{i}$ denote the bulk modulus, coefficient of thermal expansion, and volume fraction, respectively, of component i [65]. The bulk modulus and the coefficient of thermal expansion of HfV_2_ (HfV_2_O_7_) of 117 GPa [32] (56 GPa [49]) and 9.9 × 10^−6^ K^−1^ [36] (−7.2 × 10^−6^ K^−1^ [40]), respectively, were used for the calculation of $\alpha_{c}$. The resulting volume fraction dependent expansion coefficient $\alpha_{c}$ and averaged *TCE* of HfV_2_–HfV_2_O_7_ composite are plotted in Figure 1.

The coefficient of thermal expansion of the composite was calculated to reach temperature invariance ($\alpha_{c}$ = 0) at a volume fraction of HfV_2_ of around 0.25 ($\phi_{\mathrm{HfV}2O7}$ = 0.75). A *TCE* of zero was achieved for $\phi_{HfV2}$ of around 0.35 ($\phi_{HfV2O7}\;$= 0.65) with a corresponding $\alpha_{c}$ of 1.9 × 10^−6^ K^−1^, which complies with the class of very low thermal expansion materials [1]. These results not only suggest that $\alpha_{c}$ and *TCE* of this composite can individually be adjusted to thermal invariance but also that a near-zero $\alpha_{c}$ and *TCE* may be achieved simultaneously. Based on the applied data and the assumptions outlined above, we predict that the corresponding volume phase fractions of HfV_2_ (HfV_2_O_7_) are between 20 and 40 (80–60) vol.% depending on the optimization criterion. After assessing the capability of composite HfV_2_–HfV_2_O_7_ to exhibit temperature-independent properties, its synthesis is studied in the following.

### 3.2. Composite Formation by Oxidation of HfV_2_

A potential synthesis pathway for a composite has been demonstrated by oxidizing TiN thin films, where the time- and temperature-dependent formation of a TiO_2_ scale on top of TiN was observed with varying thickness, and hence phase fraction ratios [67,68]. Consequently, the first synthesis strategy to form HfV_2_–HfV_2_O_7_ composites comprises synthesis of HfV_2_ and subsequent heat treatment in air to partly oxidize HfV_2_ in an attempt to form a HfV_2_O_7_ oxide scale on top.

#### 3.2.1. Phase Formation of HfV_2_

**Figure 2:**
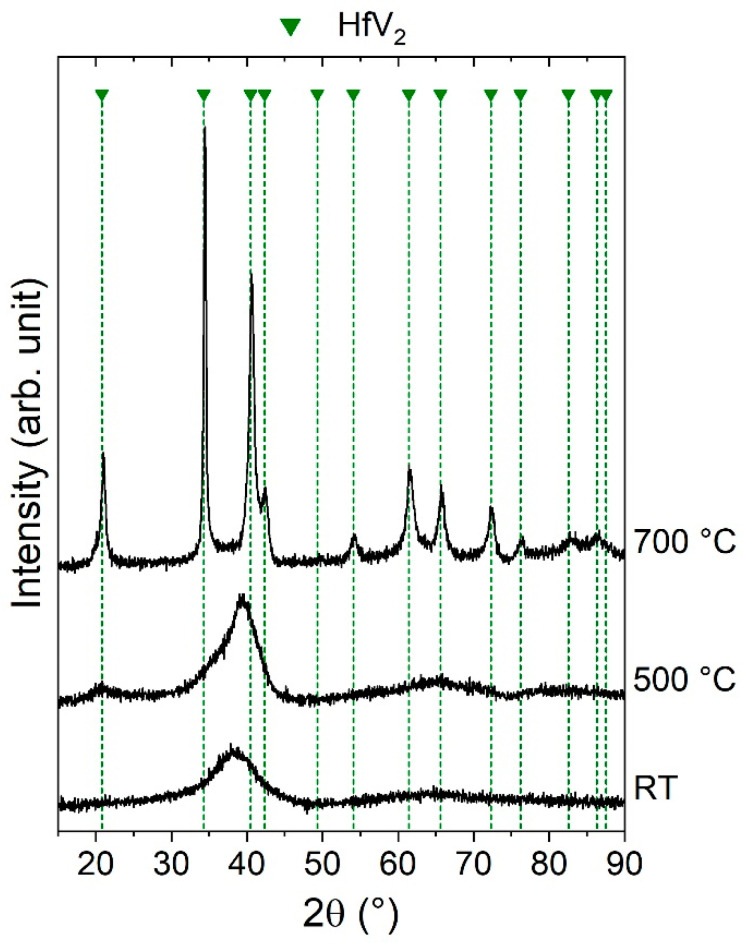
X-ray diffractograms of stoichiometric (V/Hf = 2.0) Hf-V samples synthesized without intentional heating (RT) and at synthesis temperatures of 500 and 700 °C.

First, the phase formation for co-sputtered Hf-V thin films using magnetron sputtering is described. No previous reports on the synthesis of HfV_2_ thin films by magnetron sputtering are currently available. However, the synthesis of isoelectronic and isostructural ZrV_2_ thin films is discussed in the literature [69,70,71]. EDX analysis of the as-deposited thin films shows deviations from the desired 1Hf:2V stoichiometry below ±1 at.%. The results of the structural analysis to study the influence of the deposition temperature on the phase formation are depicted in Figure 2.

A broad hump around 40° was obtained for HfV_2_ deposited without intentional heating (RT), indicating an amorphous structure, as reported for magnetron sputtered Zr–V thin films deposited without heating [71] and at 400 °C [70]. In this temperature regime, in which the adatom mobility is low, the formation of amorphous solid solution in Zr–V can be understood based on its higher stability compared to a random bcc solid solution, as demonstrated by DFT calculations [71]. It is reasonable to assume that this may also apply to Hf-V.

For Zr–V, a phase formation sequence from amorphous (400 °C) to a phase mixture of bcc V and hcp Zr (500 °C) to intermetallic ZrV_2_ (600 °C) was obtained with increasing deposition temperatures [70]. The same sequence was obtained by annealing amorphous films [70]. The intermediate formation of bcc V and hcp Zr is contradictive to the accepted equilibrium phase diagram and is mostly discussed with respect to difficult nucleation kinetics of the Laves phase structure [69,70]. However, it may also be explained by the thermodynamic instability of cubic ZrV_2_, as predicted by DFT in the ground state [71,72,73], exhibiting an energy of formation of 150 meV atom^−1^ [71], which persists at elevated temperatures. In comparison, several theoretical studies also predict cubic HfV_2_ to be energetically unstable in the ground state, reporting energies of formation between 20 and 35 meV per atom [29,74,75]. Furthermore, experiments show a transformation of cubic HfV_2_ upon cooling into an orthorhombic structure at around −160 °C [76,77] possibly due to kinetically limited decomposition into elemental V and Hf, since orthorhombic HfV_2_ also exhibits positive energy of formation [29,75]. However, for sputtered HfV_2_ at 500 °C (see Figure 2), the change in shape of the main peak at around 38° suggests the first formation of nanocrystals, and the emerging hump around 20° points towards intermetallic HfV_2_ nanocrystals, since their presence cannot be explained by hcp Hf or bcc V.

A further increase in deposition temperature to 700 °C resulted in the formation of sharp diffraction peaks which all can be assigned to cubic HfV_2_ [78]. Thus, no phase mixture of hcp Hf and bcc V, unlike for Zr–V, was observed, but a sequence from amorphous to crystalline HfV_2_ with increasing deposition temperature was obtained. Consequently, the high temperature of 700 °C required to form crystalline HfV_2_ is attributed to the kinetically limited formation of the Laves phase structure and not to an energetic instability of the cubic structure up to these temperatures. This notion is supported by theoretical predictions suggesting the energetic stabilization of cubic HfV_2_ at temperatures as low as −120 °C due to lattice vibrations [29]. For ZrV_2_, due to the considerably higher energy of formation in the ground state [71], its energetic stabilization is expected at higher temperatures, potentially explaining the discussed phase formation differences between Hf-V and Zr–V thin films. For comparison, bulk synthesis of HfV_2_ includes heat treatments at temperatures above 1200 °C [34,77,79]. The reduction in synthesis temperature to 700 °C is enabled by surface diffusion of adatoms during sputtering [80].

No indications for impurity phases based on the presented XRD results were obtained in these samples, which is ascribed to the measures outlined above. However, thin films synthesized at higher base pressures or without additional co-sputtering of Zr contained traces of Hf_3_V_3_O and HfO_2_ (not shown). This is in agreement with the observation of ZrV_3_O_3_ as an impurity phase in sputtered Zr–V thin films [70].

#### 3.2.2. Stability of HfV_2_

**Figure 3:**
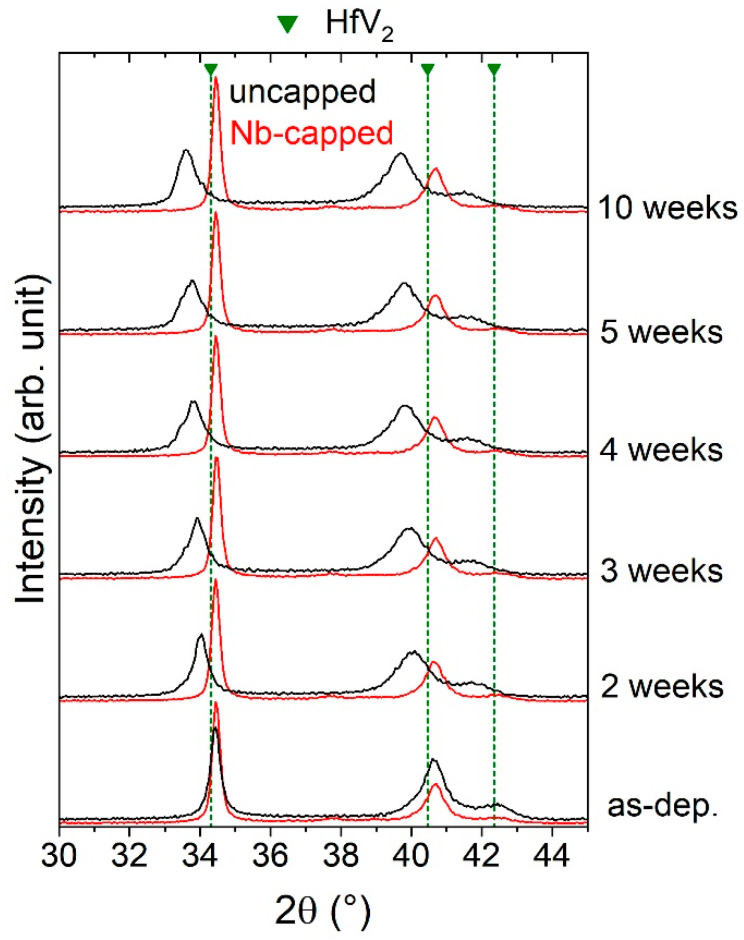
X-ray diffractograms of uncapped HfV_2_ (black) and Nb-capped HfV_2_ (red) for varying storage times in air.

As a next step, the stability of the synthesized HfV_2_ films upon air exposure is examined. For this purpose, the structure of uncapped HfV_2_ was studied as a function of the cumulated air-exposure time after removing it from the high-vacuum deposition system. The results are summarized in Figure 3.

In the as-deposited state, referring to a minimized air-exposure time of approximately 15 min, the peak positions coincide well with literature data for cubic HfV_2_ [78]. With increasing air-exposure time, a continuous peak shift to lower 2θ values is obtained, indicating an increase in the lattice parameter of the cubic structure from 7.38 to 7.51 Å (+1.8%) after four and to 7.54 Å (+2.2%) after ten weeks in air (standard deviations ≤ 0.01 Å). The obtained increase in lattice parameter may suggest continuous interstitial incorporation of impurities into the HfV_2_ lattice, which consequently also explains reported property changes in (Hf,Zr)V_2_ bulk samples after a one-year storage period [81]. HfV_2_ has been reported to act as a strong (weak) getter for hydrogen (oxygen) by dissociation of water [82].

Thin metal capping layers were shown to serve as effective oxidation barriers during air exposure at room temperature [83]. Hence, as-deposited HfV_2_ was capped with approximately 10 nm Nb due to its passivating properties [84]. The functionality of this capping layer was investigated systematically by comparing the stabilities of capped and uncapped HfV_2_ using XRD (see Figure 3). Other than a small additional peak around 38°, measured for the Nb-capped film, which is attributed to bcc Nb, no difference in phase composition between both HfV_2_ films in the as-deposited state was observed. However, in contrast to the uncapped HfV_2_ film, no peak shift with increasing air-exposure time was obtained, demonstrating that the Nb capping ensures protection of synthesized HfV_2_ against the incorporation of impurities from the ambient at room temperature.

**Figure 4:**
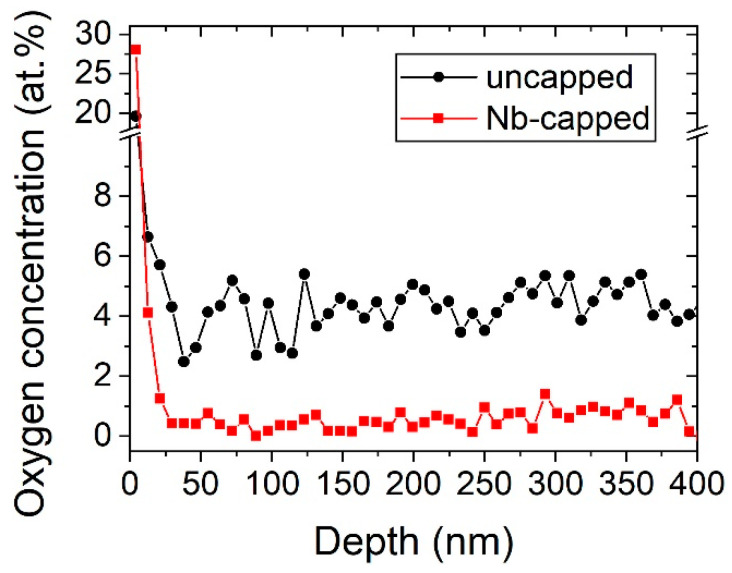
ToF-ERDA depth profile of the oxygen concentration for uncapped (black circle) and Nb-capped (red square) HfV_2_ after four weeks of air-exposure.

To identify the incorporated impurities, the chemical compositions of both samples, uncapped and Nb-capped, were analyzed four weeks after deposition using ToF-ERDA. The measured oxygen concentration as a function of the film thicknesses is presented in Figure 4. The depth scale was calculated with the atomic masses of Hf and V under the assumption of stoichiometric HfV_2_ with a density of 9.3 g cm^−3^ [32].

Uncapped HfV_2_ exhibited an averaged oxygen concentration of 4.3 ± 0.7 at.% in the bulk part of the film (neglecting the top surface oxidation) and a minor amount of hydrogen (<0.3 at.%, not shown) while the average oxygen concentration in the Nb-capped film was 0.5 ± 0.3 at.% and hydrogen was below the detection limit. Thus, the increasing lattice parameter for uncapped HfV_2_ is primarily attributed to a continuous interstitial uptake of oxygen into the cubic structure upon exposure to air. It has been shown theoretically that interstitially incorporated oxygen contributes to the energetic and mechanical stabilization of the cubic structure [29]. The oxygen concentration was measured to be constant throughout the analyzed film thickness of approximately 400 nm, suggesting high mobility of oxygen in cubic HfV_2_ already at room temperature. This may also have implications for the oxidation behavior of these films at elevated temperatures, which is discussed in the following.

#### 3.2.3. Oxidation of HfV_2_

**Figure 5:**
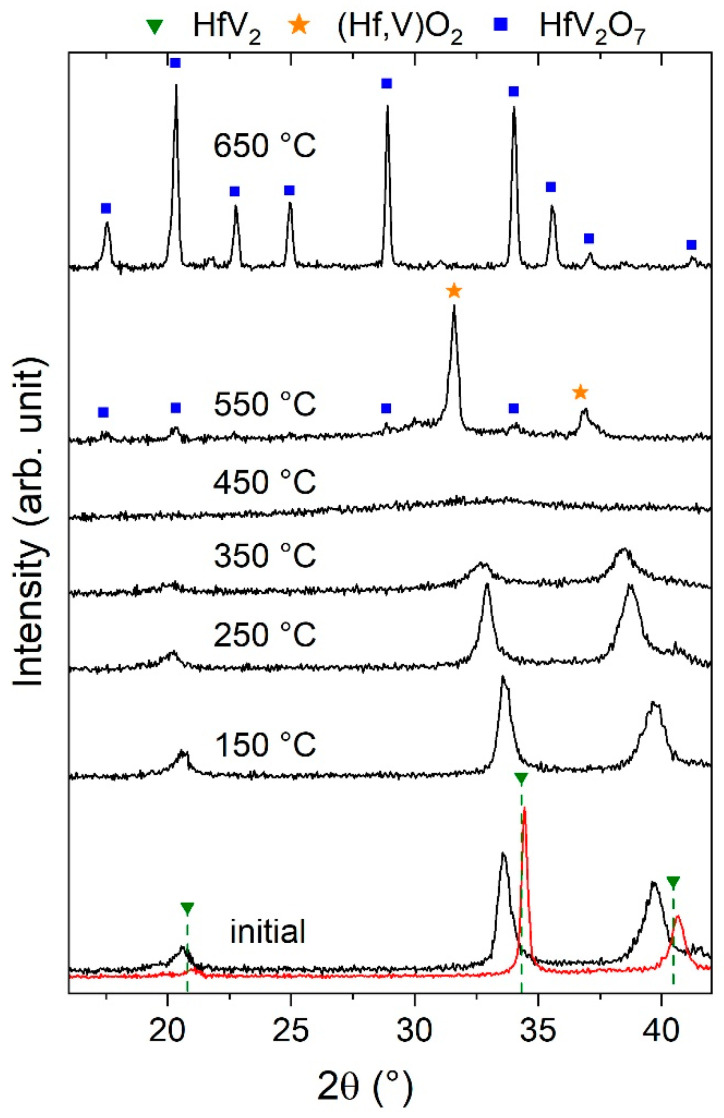
X-ray diffractograms of an uncapped HfV_2_ thin film (9 weeks after deposition) annealed at the indicated temperatures for approximately 30 min. For comparison, the diffractogram of a Nb-capped film was added (red).

One sample of uncapped HfV_2_ was cyclically annealed in air for approximately 30 min at temperatures between 150 and 650 °C in 100 °C intervals. After each annealing step, the structure of the sample was studied by XRD. The resulting diffractograms are shown in Figure 5.

In the initial state, the sample was already exposed to air for 9 weeks. As a consequence, peak positions were already shifted towards smaller 2θ values compared to the as-deposited (see Figure 3) and Nb-capped films (see Figure 5). For all annealing steps up to 350 °C, the structural development is characterized by a continuous peak shift and broadening suggesting the successive incorporation of oxygen into the cubic structure finally yielding amorphization. as observed at 450 °C. Complementary annealing experiments with additional samples at fixed temperatures of 300 and 400 °C indicate that this process is determined by kinetics and may occur already at lower temperatures (not shown).

After annealing at 550 °C, small peaks emerged which may already indicate the formation of HfV_2_O_7_ nanocrystals [41], while the two main peaks at 32° and 37° cannot be attributed to any reported equilibrium phase in the Hf-V–O materials system. The peak positions of the unknown phase fit reasonably well to a cubic (fcc-based) structure exhibiting a lattice parameter of 4.9 Å. The reported cubic structures in the Hf-V–O systems, VO (Fm$\bar{3}$m) [85] and HfO_2_ (Fm$\bar{3}$m) [86], can be excluded due to the difference in the lattice parameters. Furthermore, the formation of a hypothetic cubic Hf_1−*x*_V*_x_*O monoxide appears unlikely based on conducted DFT studies, predicting a lattice parameter between 4.19 and 4.58 Å for *x* = 0 and *x* = 1, respectively. Nevertheless, the experimentally obtained lattice parameter lies within the predicted lattice parameter range of a previously unreported Hf_1-*x*_V*_x_*O_2_ dioxide (Fm$\bar{3}$m) which is between 5.08 and 4.74 Å for *x* = 0 and *x* = 1, respectively. Hence, the formation of a metastable ternary (Hf,V)O_2_ phase is assumed henceforth. A high V solubility in cubic HfO_2_ has also been reported elsewhere [87]. At 650 °C, the formation of XRD phase pure HfV_2_O_7_ is evident [41].

The observation that the first crystallites of HfV_2_O_7_ may form at temperatures of 550 °C while oxygen is highly mobile in HfV_2_ already at room temperature suggests that the formation of HfV_2_–HfV_2_O_7_ composites is not feasible using conventional annealing experiments in air. The oxygen mobility in HfV_2_ at the formation temperature of crystalline HfV_2_O_7_ appears to be critical for the endeavor of HfV_2_–HfV_2_O_7_ composite formation. However, lowering of the synthesis temperature of crystalline HfV_2_O_7_ by using non-equilibrium-based synthesis approaches appears promising and is discussed in the following.

### 3.3. Composite Formation by Sputtering of Bilayered HfV_2_–HfV_2_O_7_

#### 3.3.1. Phase Formation of HfV_2_O_7_

**Figure 6:**
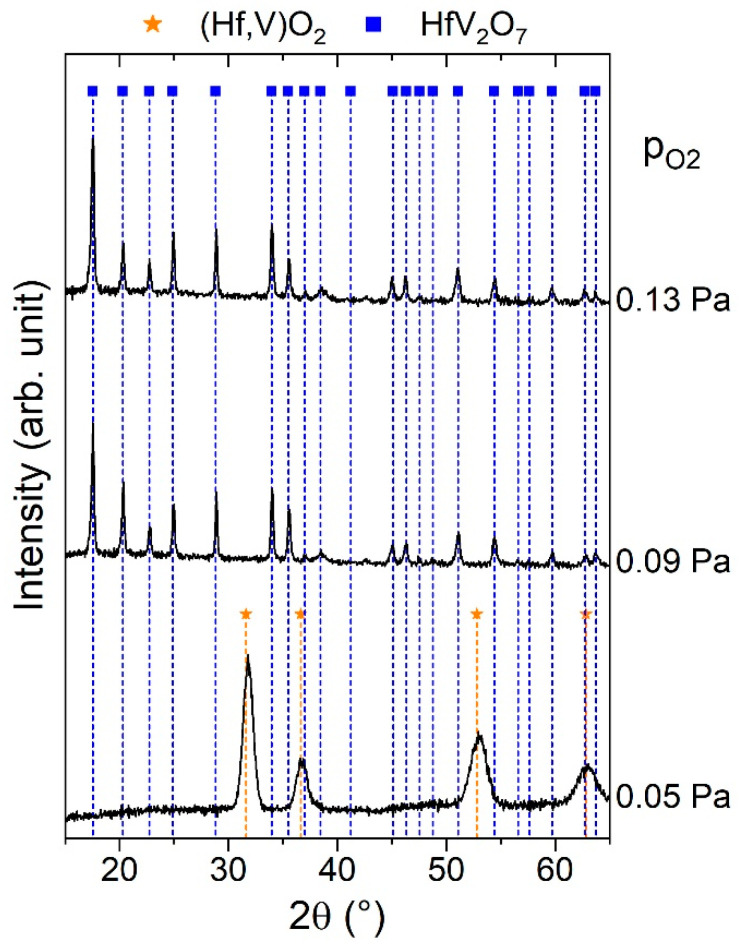
X-ray diffractograms of reactively sputtered Hf-V–O (V/Hf = 2) thin films deposited at 450 °C with varying O_2_ partial pressure.

The formation of crystalline HfV_2_O_7_ at a substrate temperature of 350 °C using reactive magnetron sputtering has been demonstrated previously [49], but the effect of the O_2_ partial pressure has not been investigated yet. However, for the synthesis of bilayered HfV_2_–HfV_2_O_7_ by magnetron sputtering, the O_2_ partial pressure during the synthesis of HfV_2_O_7_ is expected to be decisive for the purity of the underlying HfV_2_ film, due to its high oxygen affinity. Hence, the influence of the O_2_ partial pressure on the phase formation of HfV_2_O_7_ was studied systematically for a synthesis temperature of 450 °C. The results of the structural analysis of synthesized thin films, exhibiting a V–Hf ratio of 2.0 based on EDX, are shown in Figure 6.

The thin film sputtered at an O_2_ partial pressure of 0.05 Pa exhibits broad peaks at around 32°, 37°, 53°, and 63°. The peak positions coincide well with the ones of the cubic (Hf,V)O_2_ structure observed during annealing of HfV_2_ at 550 °C (see Figure 5). Cubic HfO_2_ usually constitutes the high-temperature polymorph, which is stable above 2600 °C at ambient pressure [86] but can be stabilized down to room temperature by V doping in specific atmospheres (oxidation state ≤ V^4+^) [88]. Furthermore, cubic HfO_2_ thin films form by ion beam assisted deposition under oxygen deficiency and substrate cooling [89]. Thus, it is assumed that the formation of cubic (Hf,V)O_2_ obtained for the lowest O_2_ partial pressure is triggered by oxygen deficiency. This notion is supported by the fact that the increase in the O_2_ partial pressure from 0.05 to 0.09 Pa resulted in a sixfold decrease in deposition rate (with respect to the mass gain), which indicates a more pronounced poisoning of the target racetrack. For O_2_ partial pressures of 0.09 and 0.13 Pa, all obtained peaks can be assigned to the HfV_2_O_7_ structure [41], indicating the formation of phase pure HfV_2_O_7_ based on XRD.

#### 3.3.2. Thermal Expansion of Sputtered HfV_2_O_7_

**Figure 7:**
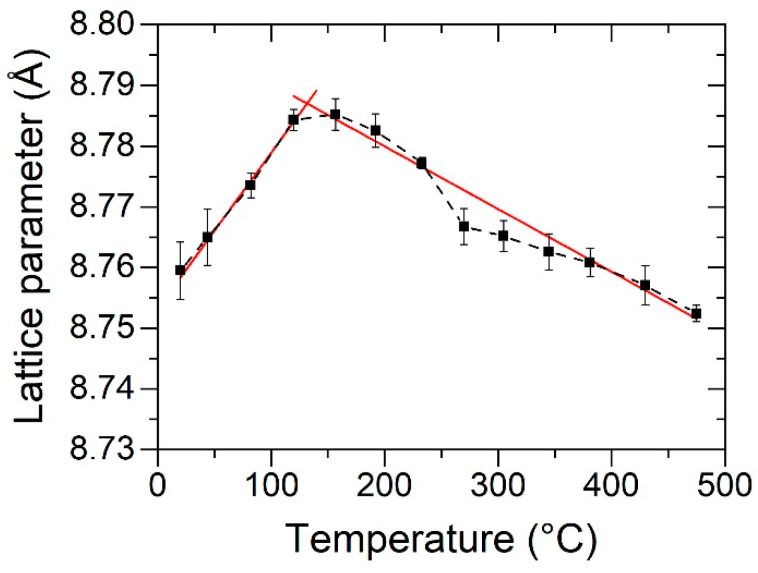
Lattice parameter of synthesized cubic HfV_2_O_7_ as a function of annealing temperature obtained by X-ray diffraction. A linear fit within the positive and negative thermal expansion range, respectively, was added (red).

While numerous NTE materials have intensively been studied in bulk, studies on thin films are scarce [42,43,44,45]. To verify the NTE behavior of the synthesized HfV_2_O_7_ thin films, temperature-dependent in situ XRD measurements were performed to measure the change in the lattice parameter upon heating. The results are shown in Figure 7.

A continuous increase in the lattice parameter between 20 and 120 °C was measured, whereas between 155 and 475 °C the lattice parameter decreased, indicating NTE. The obtained transition from PTE to NTE is in agreement with reported transition temperatures of HfV_2_O_7_ being around 120–130 °C produced by solid-state reaction synthesis approaches [40,41,64]. Additional wafer curvature measurements confirm this transition, as indicated by a change from increasing compressive to increasing tensile stress upon heating (not shown). The NTE properties of HfV_2_O_7_ originate from its openly-packed network structure consisting of octahedral HfO_6_ and tetrahedral VO_4_ units, constituting quasi-rigid building blocks that are interconnected by corner-sharing oxygen atoms [4,90,91]. Three of four oxygen atoms of the VO_4_ tetrahedron are shared with neighboring HfO_6_ octahedra, while one is shared with another VO_4_ tetrahedron, thereby forming a V_2_O_7_ group [92]. The lattice symmetry (space group: Pa$\bar{3}$) restricts these O_3_V–O–VO_3_ bonds to retain an angle of 180°, allowing for transverse vibrations that may give rise to NTE [4,93]. The loss of the NTE properties at lower temperatures is related to structural transitions. HfV_2_O_7_ transforms upon cooling via an intermediate incommensurate structure to a 3 × 3 × 3 superstructure [41,64]. It has been shown for isostructural ZrV_2_O_7_ that the majority of the O_3_V–O–VO_3_ linkages bend away from 180° in the 3 × 3 × 3 superstructure [94,95,96]. However, the additional weak reflections of the HfV_2_O_7_ superstructure [97] could not be resolved by XRD in this work. Based on the presented data, the linear coefficients of thermal expansion were determined to be (2.8 ± 0.5) × 10^−5^ K^−1^ (20 °C ≤ T ≤ 120 °C) and (−9.9 ± 0.9) × 10^−6^ K^−1^ (155 °C ≤ T ≤ 475 °C), respectively, agreeing reasonably well with the reported linear coefficients of thermal expansion of 2.5 × 10^−5^ K^−1^ [64] and −7.2 × 10^−6^ K^−1^ [40]. After establishing a synthesis recipe for NTE material HfV_2_O_7_ that is characterized by a low required O_2_ partial pressure, the way is paved for the synthesis of bilayered HfV_2_–HfV_2_O_7_ composites using a two-staged sputtering process.

#### 3.3.3. Phase Formation of HfV_2_–HfV_2_O_7_ Bilayers

**Figure 8:**
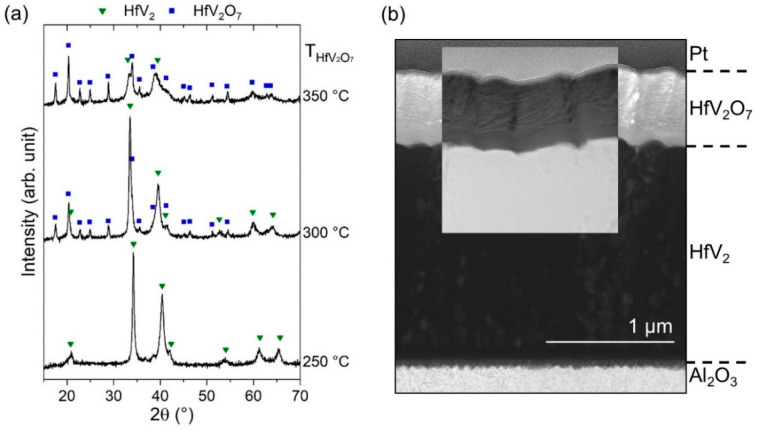
(**a**) Diffractograms of magnetron sputtered HfV_2_–HfV_2_O_7_ bilayers. The synthesis temperature *T* for HfV_2_O_7_ on HfV_2_ has been varied between 250 and 350 °C. (**b**) Scanning transmission electron microscopy bright-field with inset high-angle annular dark-field image of HfV_2_–HfV_2_O_7_ cross-section with HfV_2_O_7_ having been synthesized at 300 °C.

Besides a low O_2_ partial pressure during reactive sputtering of HfV_2_O_7_, the synthesis temperature for HfV_2_O_7_ is decisive for avoiding substantial oxygen incorporation into HfV_2_. Hence, the influence of synthesis temperature of HfV_2_O_7_, which was varied between 250 and 350 °C, on the phase formation of HfV_2_–HfV_2_O_7_ composite was investigated by maintaining a low O_2_ partial pressure of 0.09 Pa (see Figure 6). While a Nb passivation layer ensures protection of HfV_2_ in air at room temperature (see Figure 3), additional bilayer depositions revealed that the deposition of a Nb interlayer, separating the two layers in the composite, does not prevent oxygen incorporation in HfV_2_ during reactive sputtering of HfV_2_O_7_ (not shown) and is therefore not discussed further. The results of the structural analysis are shown in Figure 8.

For a synthesis temperature of 250 °C, peaks that are attributed to HfV_2_ are barely shifted [78] indicating a low concentration of interstitially incorporated oxygen into the structure during the reactive sputtering process of HfV_2_O_7_. However, no distinct HfV_2_O_7_ peaks were obtained indicating an amorphous or nanocrystalline structure. It is expected that amorphous HfV_2_O_7_ does not exhibit NTE. While crystalline ZrW_2_O_8_ exhibits NTE in its entire stability range [38,39], PTE was obtained for amorphous ZrW_2_O_8_ films synthesized by reactive sputtering [42].

An increase in synthesis temperature to 300 °C results in more pronounced oxygen incorporation in HfV_2_, as indicated by the increasing peak shift, which corresponds to the shift obtained for a 10-weeks exposure time of uncapped HfV_2_ to air (see Figure 3). Besides, peaks of crystalline HfV_2_O_7_ emerge, demonstrating the first synthesis of a crystalline HfV_2_–HfV_2_O_7_ composite. The effect of minute concentrations of interstitially solved oxygen on the thermoelastic anomaly of HfV_2_ has not been studied yet and is hence proposed for future investigations. Figure 8b shows the corresponding film cross-section studied by STEM. The image suggests the formation of a bilayered structure with a defined interface separating the HfV_2_ bottom layer (1.7 µm) from HfV_2_O_7_ (0.5 µm) top layer. Based on the atomic number (*z*) contrast using high-angle annular dark-field imaging, the distinct formation of HfV_2_ (high *z*) and HfV_2_O_7_ (low *z*) is supported. The deposition rates for HfV_2_ and HfV_2_O_7_ were determined to be approximately 22 and 5 nm min^−1^, respectively. Thus, tuning the volume phase fractions to be in line with the proposed ones (see Figure 1) can simply be achieved by adjusting the deposition times for the individual layer. However, further investigations are required to evaluate whether these bilayered composite films allow for accurate determination of their integral temperature coefficient of elasticity and coefficient of thermal expansion. A further increase in the synthesis temperature of HfV_2_O_7_ to 350 °C reveals the onset of amorphization of HfV_2_ due to oxygen incorporation, as indicated by the pronounced peak shifting and broadening.

## 4. Conclusions

The HfV_2_–HfV_2_O_7_ composite has been proposed as a material with temperature-independent thermophysical properties due to the combination of the anomalously increasing thermoelastic constants of HfV_2_ and the negative thermal expansion of HfV_2_O_7_. Both the temperature coefficient of elasticity and the coefficient of thermal expansion were predicted to be near zero for a phase fraction of approximately 30 vol.% HfV_2_ and 70 vol.% HfV_2_O_7_.

Two synthesis pathways for HfV_2_–HfV_2_O_7_ composites were studied: (1) annealing of magnetron sputtered HfV_2_ thin films in air to form a HfV_2_O_7_ oxide scale on the thin film surface and (2) magnetron sputtering of HfV_2_O_7_/HfV_2_ bilayers. The onset of the oxidation behavior of HfV_2_ thin films is characterized by continuous interstitial incorporation of oxygen, occurring already at room temperature, finally yielding amorphization. Crystalline HfV_2_O_7_ forms at 550 °C. The high oxygen mobility in HfV_2_ is suggested to inhibit the formation of crystalline HfV_2_–HfV_2_O_7_ composites by annealing HfV_2_ in air. Reducing the formation temperature of crystalline HfV_2_O_7_ down to 300 °C using reactive magnetron sputtering enables the synthesis of a crystalline bilayered HfV_2_–HfV_2_O_7_ composite. The NTE properties of HfV_2_O_7_ were verified for monolithic magnetron sputtered HfV_2_O_7_ thin films using temperature-dependent in situ X-ray diffraction.
